# “Sex without fear”: exploring the psychosocial impact of oral HIV pre-exposure prophylaxis on gay men in England

**DOI:** 10.1186/s12981-023-00568-2

**Published:** 2023-11-14

**Authors:** Rosalie Hayes, Will Nutland, Michael Rayment, Sonali Wayal, Vanesa Apea, Amanda Clarke, Alan McOwan, Ann Sullivan, Monica Desai, Andrew Jajja, Brian Rice, Rob Horne, Sheena McCormack, Mitzy Gafos

**Affiliations:** 1 Wolfson Institute of Population Health, Queen Mary University of London, 58 Turner Street, London, E1 2AB UK; 2 Prepster, UK; 3 Directorate of HIV and GU Medicine, Chelsea and Westminster Hospital NHS Foundation Trust, London, UK; 4 Institute for Global Health, Mortimer Market Centre, University College London, London, UK; 5 Ambrose King Centre, Barts Health NHS Trust, London, UK; 6 Department of HIV, Sexual Health and Contraception, Royal Sussex County Hospital, University Hospitals Sussex NHS Trust, Brighton, UK; 7 STI & HIV Division, Blood Safety, Health Security Agency, London, UK; 8 Faculty of Public Health and Policy, London School of Hygiene and Tropical Medicine, London, UK; 9 School of Health and Related Research, University of Sheffield, Sheffield, UK; 10 School of Pharmacy, University College London, London, UK; 11 Medical Research Council Clinical Trials Unit, University College London, London, UK

**Keywords:** HIV prevention, Pre-exposure prophylaxis, Gay bisexual and other men who have sex with men, Psychosocial impact, Sexual anxiety

## Abstract

Gay, bisexual, and other men who have sex with men (GBMSM) experience a high prevalence of psychosocial health problems, such as harmful substance use and depression, as well as being disproportionately affected by HIV. HIV Pre-Exposure Prophylaxis (PrEP) may provide psychosocial benefits beyond its intended purpose of reducing HIV infection. We explore the psychosocial impact of oral PrEP use on gay men in England using qualitative data from the PROUD study. From February 2014 to January 2016, semi-structured in-depth interviews were conducted with 40 gay men and one trans woman. Participants were purposively recruited based on trial arm allocation, adherence, and sexual risk behaviours. By removing HIV risk from sex, PrEP improves users’ wellbeing by reducing HIV-related anxiety and internalised stigma and increasing HIV prevention self-efficacy, sexual pleasure, and intimacy. In turn, these psychological changes may influence behaviour in the form of greater sexual freedom, reduced harmful drug use, and more protective sexual health behaviours. However, PrEP may create internal conflict for some gay men, due to its disruption of social norms around condom use and its perceived influence on their sexual behaviour leading to reduced condom self-efficacy. These findings provide a baseline of PrEP’s psychosocial impact amongst some of the first PrEP users in England and supports calls to consider the psychosocial impact of PrEP in prescribing guidelines.

## Introduction

Gay, bisexual, and other men who have sex with men (GBMSM) experience a higher global prevalence of psychosocial health problems including harmful substance use, intimate partner violence (IPV), and mental health issues such as depression and anxiety, compared to heterosexuals [1–3]. These disparities have been attributed to the higher stress levels experienced by sexual minorities due to internalised, anticipated and experienced prejudice and discrimination on the basis of their sexuality [4].

GBMSM are also disproportionately affected by HIV [5], and a psychosocial stressor that is heightened for GBMSM is HIV-related anxiety during sex. The Hunter Alliance for Research and Translation found across multiple studies that 25–39% of GBMSM say they think about HIV in the day-to-day either most or all the time, and 29–46% say they think about HIV most or all the time during sex [6]. Golub (2018) describes this as a “psychological tragedy” [7] burdening GBMSM since the 1980s emergence of HIV, where thoughts of risk and fear intrude during moments that should be focused on intimacy, pleasure, and fulfilment. While HIV-related anxiety may be omnipresent for many GBMSM, a heightened awareness of HIV risk does not always translate to consistent condom use [8]. There are other factors which can increase the likelihood of engaging in condomless sex, both positive – such as a desire for greater sexual pleasure, sense of intimacy, and emotional closeness with sexual partners – and negative – including psychosocial trauma and internalised stigma [8]. The presence of one or more of these factors may be sufficient to override HIV-related anxiety and facilitate engagement in condomless sex.

HIV Pre-exposure Prophylaxis (PrEP) offers effective HIV prevention for those who aren’t able to consistently use condoms. PrEP refers to the use of antiretroviral therapy (ART) by HIV-negative people before and during periods of exposure to HIV (for example, via condomless sex) to prevent acquisition of HIV. Tenofovir disoproxil fumarate combined with emtricitabine (TDF/FTC) was the first drug formulation approved as PrEP [9]. Until 2020, the World Health Organization only endorsed oral PrEP in the form of tablets taken daily or episodically; delivery methods now also include the dapivirine vaginal ring and long-acting injectable cabotegravir [10, 11].

The PROUD (PRe-exposure Option for reducing HIV in the UK: immediate or Deferred) trial demonstrated in 2015 that the inclusion of oral PrEP in the sexual health package offered to GBMSM and trans women in sexual health clinics in England reduced HIV acquisition by 86%, with no infections observed among participants taking PrEP at the likely time of exposure [12]. Baseline data from PROUD showed that the offer of oral PrEP attracted participants with a higher risk of HIV acquisition than the general population of GBMSM in England [13], and high rates of associated risk factors, including previous and incident STIs, previous use of PEP, drug use (particularly of chemsex-associated drugs), higher-risk sexual behaviours, depression, and intimate partner violence [13, 14]. More recent research has indicated that PrEP is contributing to a population-level reduction in HIV incidence among GBMSM in England and elsewhere [15–17].

Anecdotal evidence began to emerge as early as 2014 that PrEP was having an impact on GBMSM’s health and wellbeing in a manner that went far beyond its primary function of preventing HIV transmission [18]. Subsequently, PrEP use has been associated with a reduction in HIV-related anxiety and internalised homophobia, and an increased sense of sexual satisfaction, intimacy, and self-efficacy [19–23]. However, PrEP users may also face stigma from others who perceive PrEP as facilitating a deterioriation in sexual responsibility among GBMSM, typified by the derogatory term ‘Truvada whores’ [24, 25] (Truvada (Gilead Sciences, Foster City, CA, USA) is the branded name for TDF/FTC). Among GBMSM, the presence of these attitudes reflect not only internalised homophobia, but also the mindset of sexual prudence which replaced gay sexual liberation during the early days of the HIV epidemic. Consequently, condomless sex has become intertwined with problematic notions of uncleanliness, contamination, and ‘sluttiness’ [26].

Given the implications of these issues for uptake and continuation, understanding the psychosocial impact of PrEP use among is therefore important for maximising its individual and public health benefits. Thus far, there has been limited research considering the broader psychosocial impact of PrEP in the UK. A cross-sectional survey led by PrEPster and Public Health England asked PrEP users if PrEP had a positive or negative impact on their life, with open text responses indicating that experiences included reduced stress and anxiety, but this was not explored in detail [27].

‘Psychosocial’ factors can be understood as an intermediary (meso-level) bridge between macro-level social structures and the micro-level individual, such as support from social networks, control at work or in the home, security, and autonomy [28]. Martikinainen et al. (2002) describe a psychosocial explanation of health as one in which macro- and meso-level social processes lead to perceptions and psychological processes at the individual level, which in turn influence health through biological responses to stress or changes in behaviours and lifestyles [28].

In this paper, we utilise this understanding of psychosocial impact to explore how the use of oral PrEP influences the interactions between meso-level psychosocial factors and individual psychological factors, and in turn how these factors affect health through emotions and behaviour.

## Methods

The PROUD study was designed to evaluate the effectiveness of daily oral TDF/FTC in preventing HIV transmission amongst GBMSM and trans women in England and understand its impact on sexual behaviour and STI transmission. It was a multi-centre, open label, randomised design in which participants were allocated 1:1 to immediate or deferred (after 12 months) offer of oral PrEP as part of an overall HIV risk reduction package. Participants were recruited from November 2012 to April 2014 at 13 sexual health clinics in England. Only HIV-negative GBMSM or trans women who reported condomless anal sex in the last three months and anticipated it again in the next three months were eligible to participate. PROUD was initially designed as a pilot study to inform the design of a larger trial. However, after its interim results indicated that oral PrEP is highly effective at reducing HIV incidence, in October 2014 the trial steering committee recommended that all participants should be offered oral PrEP [12].

From February 2014 to January 2016, PROUD participants at clinics in London, Sheffield, Manchester and Brighton were purposively sampled by the qualitative study team to take part in semi-structured in-depth interviews (IDIs). We aimed to select 44 participants based on trial arm allocation (immediate or deferred), self-reported adherence amongst participants in the immediate arm (high or medium/low), and changes in self-reported sexual risk behaviour since baseline based on number of partners and condom use (increased risk or same/decreased risk). In September 2015, these criteria were amended to select participants based on current self-reported risk behaviour to identify more variability by risk behaviour (low, medium or high risk based on highest quartile of partners and condomless sex and lowest quartile of partners and condomless sex). To investigate specific topics such as seroconversion or declining the offer of oral PrEP, we intended to purposefully select up to six additional participants. An interview guide was designed with input from the social science advisory committee and included questions about participants’ attitudes towards oral PrEP, and its influence on their self-perception and sex lives. IDIs were conducted in English by researchers independent of the study clinic team and each interview lasted around 45–75 min. Participants provided written informed consent and were not compensated.

We used a reflexive thematic analysis approach, as elaborated by Braun and Clarke [29], which is suited to describing the lived experiences of socially marginalised groups [30]. The transcripts were coded in Nvivo 12 (Lumivero, Burlington, MA, USA) using a combination of ‘theory-driven’ deductive reasoning and ‘data-driven’ inductive reasoning [31]. Deductive coding was informed by the research question and a psychosocial explanation of health; in addition to the linear process described by Martikainen et al. (2002), we were also interested in the interplay and feedback loops between levels. Inductive coding was used to generate themes from the data which reflected the participants’ perspectives, experiences and contexts situated within these domains. Themes were developed based on “particular patterns of shared meaning… united by a core concept” [29] (p5). They were then checked against the existing literature to ensure they were credible.

In our findings, we use the term ‘gay men’ as although we intended to include a broader spectrum of GBMSM, all IDI participants identified as gay.

The PROUD study protocol was approved by London Bridge Research Ethics Committee, the Medicines and Healthcare products Regulatory Agency and each of the 12 participating Hospital Trusts (listed in acknowledgements). The trial is registered with ISRCTN (Number ISRCTN94465371) and ClinicalTrials.gov (NCT02065986). The study protocol, including the Participant Information Sheet (PIS) and Informed Consent Form (ICF), and the in-depth interview PIS, ICF and interview guide, are available on the study website (www.proud.mrc.ac.uk).

## Results

**Fig. 1:**
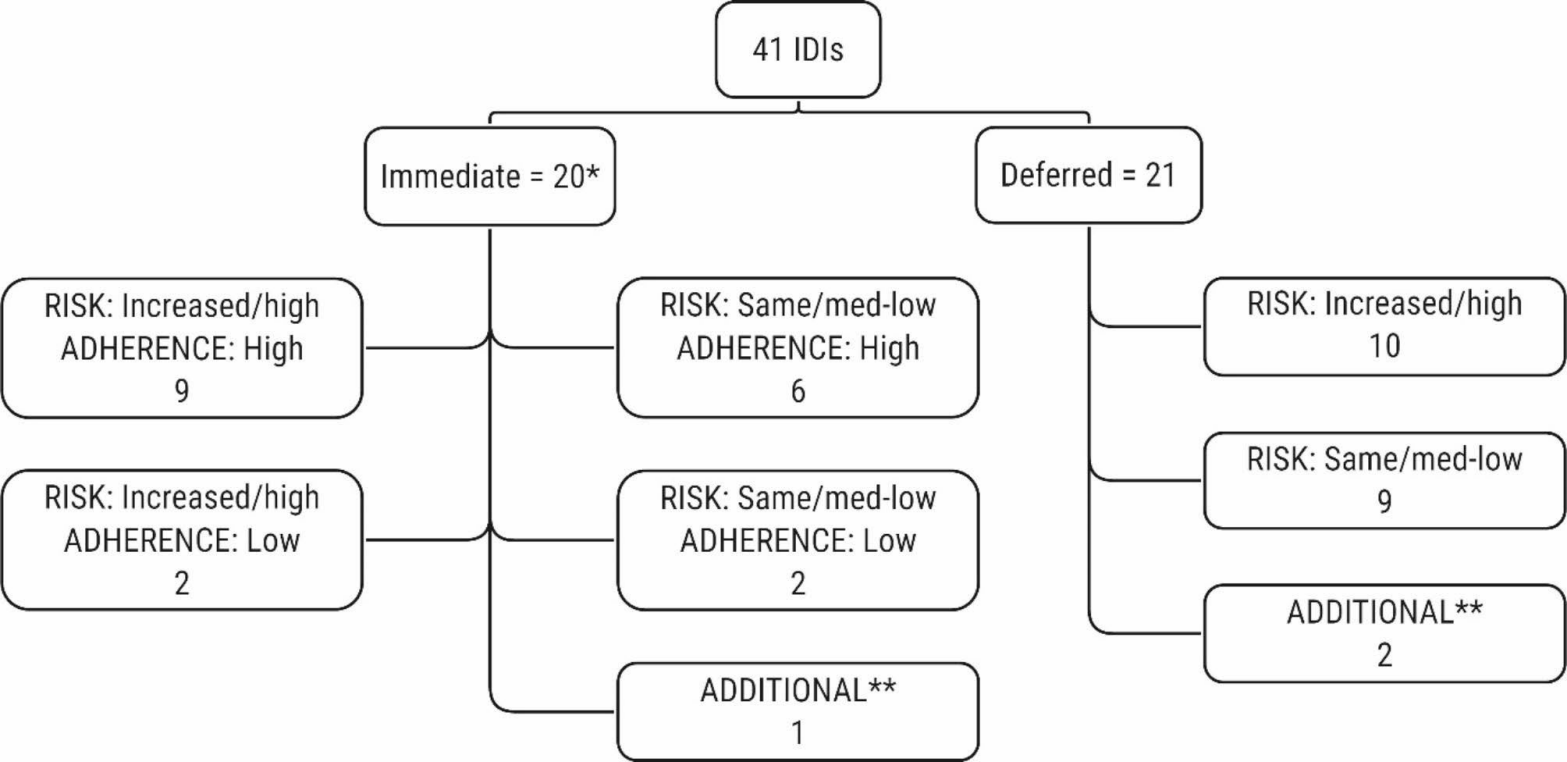
Purposeful selection of participants for IDIs based on trial arm allocation, risk behaviour and adherence * One participant co-enrolled and in the main trial analysis is treated as being in the deferred arm to which they were originally allocated. However, they are treated as being in the immediate arm here as this was how they were selected for IDI ** Additional interviews were conducted with a trans woman, a person who seroconverted during the study, and a participant who decided not to start PrEP after the deferral period

Forty-one PROUD participants were interviewed; 38 selected equally from the immediate and deferred groups and three additional interviews to explore underrepresented topics, including interviews with a trans woman, a person who seroconverted during the study, and a participant who decided not to start PrEP after the deferral period (Fig. 1).

At the time of interview, 33 out of 41 participants said they were or had been using oral PrEP. Thirty participants had been prescribed oral PrEP through PROUD and a further three participants had accessed oral PrEP privately: one had purchased oral PrEP in the USA and taken it prior to joining the study, and two had used oral PrEP during their deferred period of the trial (one by using the TDF/FTC from post-exposure prophylaxis [PEP] and the other by using TDF/FTC from an HIV-positive partner). Two participants prescribed oral PrEP in PROUD had discontinued it by the time of their IDI - one due to side effects and the other due to entering a monogamous relationship.

Duration of oral PrEP use ranged from one week to 32.8 months, with a mean of 14.3 months. Demographic characteristics of interviewed participants are presented in Table 1. Baseline data collection on sexual behaviour showed that, in line with the eligibility criteria, all IDI participants had engaged in condomless sex (39 as passive partner and 40 as active partner) in the 90 days prior to enrolling in the trial. The median number of anal sex partners at enrolment was 10, with an interquartile range of 3 to 20. Both the demographic and sexual behaviour data are also presented in qualitative studies analysing the same data published elsewhere [32, 33].

**Table 1:** Participant demographics at enrolment for IDIs (n = 41)

Demographics	N
*Median age (interquartile range)*	37.4 (31.9, 42.7)
*Clinic of enrolment*	
London Sheffield Manchester Brighton	24 9 5 3
*Ethnicity*	
White Black and Minority Ethnicities (BAME)^a^	34 7
*Place of birth*	
UK Outside of UK^b^	26 15
*University educated*	
Yes	25
No	16
*Employed*	
Yes No	36 5
*In a relationship*	
Yes No	17 24
*Sexuality*	
Gay Bisexual	40 1
*Gender*	
Cis male Trans female	40 1
*Symptoms of depression*	
Yes No	6 35
*Engagement in chemsex in the past 3 months*	
Yes^c^ No	13 28
*PEP use in last year*	
Yes^d^ No	14 26
*Self-reported STI in last year* ^e^	
Yes No	18 21

a BAME ethnicities include Pakistani, Hispanic, Arabic, and mixed ethnicity

b Outside of UK includes Australia, South America, South Africa, and the rest of Europe (1 missing)

c Chemsex use includes 12 participants using GHB, 9 using mephedrone, and 7 using crystal meth

d PEP use excludes one missing response; six participants used PEP more than once

e STIs in last year excludes two participants with missing responses

**Fig. 2:**
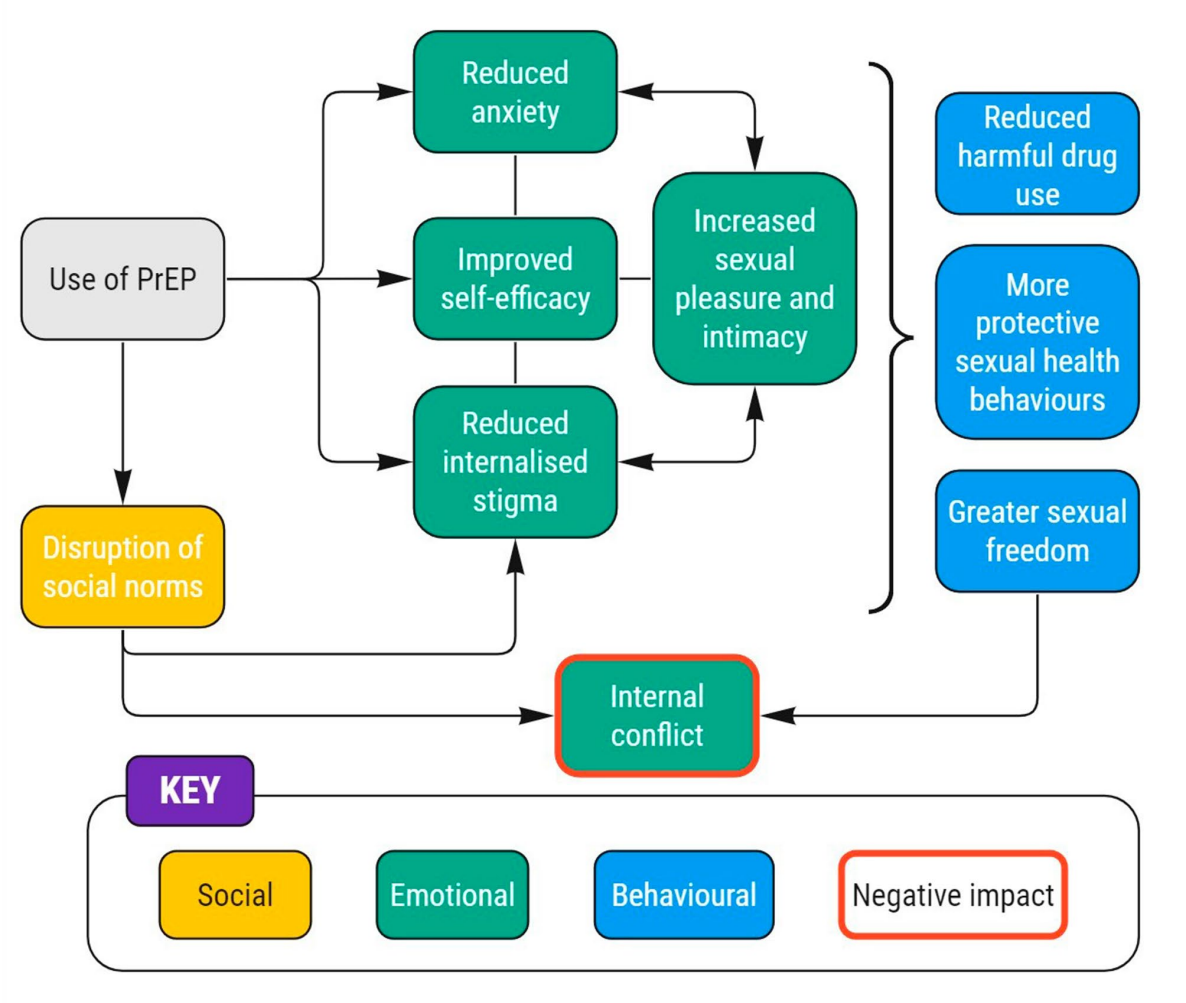
Psychosocial impact of oral PrEP use among PROUD participants

The analysis generated eight themes, as depicted in Fig. 2, which can be categorised as relating to an impact on emotions and on behaviours. As the arrows in Fig. 2 illustrate, there was considerable interplay between psychological and behavioural impacts which are explored further within each theme.

### Psychological impact of PrEP

#### Reduced HIV-related anxiety

Nearly all participants mentioned that PrEP had reduced HIV-related anxiety both during and after sex, regardless of their risk level, age, or any other characteristic. The terms ‘peace of mind’, ‘reassurance’, ‘safety’ and ‘protection’ were regularly used by participants. For example, these participants described what they most liked about PrEP:

“It just takes away any possible worry afterwards, it’s just this protective bubble there” (Deferred, decreased risk, on PrEP, Brighton, aged 35–39).

“The best part of it actually is sex without fear, it really is” (Deferred, low risk, on PrEP, Manchester clinic, aged 25–29).

For many this effect was substantial, with participants describing the reduction in anxiety as ‘a feeling of relief’, ‘a weight lifted’, and ‘life-changing’. For some, this related to growing up during the early days of the HIV epidemic. However, younger participants also described how fear of HIV acquisition affected their sex lives:

“Before PrEP I’d have sex with condoms, it was just the way it was, but then I’d still have that extra level of anxiety if I got a cold afterward… in the past I would freak out about things and [PrEP] just gives me that extra level of security.” (Deferred, medium risk, on PrEP, Brighton clinic, aged 25–29).

Most participants attributed this reduction in anxiety to the additional security they felt PrEP gave them in reducing their risk of acquiring HIV or consequently passing it on to others. A few also attributed their reduced anxiety to more regular testing in addition to the added protection provided by PrEP:

“Regular testing provided me an extra level of worry-free-ness basically as well as the Truvada itself.” (Immediate, high risk, on PrEP, Sheffield clinic, aged 30–34).

Some participants in serodiscordant relationships highlighted that their PrEP use had the secondary effect of reducing anxiety for their HIV-positive sexual partners, either regular or casual. For one participant, PrEP provided the additional reassurance his partner, who was undetectable, needed to feel comfortable having condomless sex with him:

“It’s an absolutely terrifying thing for him to think that he might infect me. It’s his absolute worst nightmare… So, we’ve tried to be extra extra safe, and [PrEP] is the extra safe barrier if the condoms aren’t there.” (Immediate, increased risk, on PrEP, London clinic, aged 35–39).

Notably, participants did not have the same level of anxiety about STIs, which were largely seen as a treatable problem. A small number of participants highlighted that they were concerned about Hepatitis C, which mostly pertained to their use of sex toys and practices such as fisting. However, in most cases concerns relating to STIs were present prior to their use of oral-PrEP since participants were already exposing themselves to potential risk and this had not changed as a result of PrEP use.

### Increased HIV prevention self-efficacy

Many participants reported increased self-efficacy, describing PrEP as making them feel more ‘responsible’, ‘mature’, and ‘in control’ of their sexual health and sexual decision-making. Several participants said they no longer felt they were having to rely on their sexual partners being honest about their HIV status and instead were able to take responsibility for their own health:

“It does give me that extra peace of mind, I do feel in control, I feel that I’m not solely responsible in believing other people.” (Deferred, high risk, on PrEP, Manchester clinic, aged 35–39).

Many participants described how, prior to PrEP use, the conflict between their sexual desire to have condomless sex and their fear of acquiring HIV reduced their self-efficacy for HIV prevention. A small number of participants experienced this to such a degree that they felt it was inevitable that they would acquire HIV. By aligning HIV risk reduction with their sexual needs and desires, PrEP offered them an opportunity for agency over their sexual health:

“I thought I was likely to carry on putting myself at risk so, although I had an attitude where I didn’t care very much what happened, I thought well I don’t want to become positive and make life even more difficult than it is already, so I was glad to go on [PrEP].” (Immediate, decreased risk, on PrEP, London clinic, aged 45–49).

Practical aspects of taking oral PrEP also contributed to increased HIV prevention self-efficacy. Several participants highlighted that the choice to protect themselves from HIV was easier with daily oral PrEP than it was with condoms, since it is not coitally-dependent:

“It’s a decision I make when brushing my teeth not when I’m in the throes of passion, that’s a decision I can make every day, very clearly with no influence, no awkwardness, no risk of changing the situation, it’s there in the medical cabinet, it’s a no brainer. Using a condom before that, using a condom reliably was hit and miss for me, and this is a much easier decision to make, that I make alone.” (Immediate, high risk, on PrEP, London clinic, aged 40–44).

### Reduced internalised stigma

Many participants expressed that PrEP had reduced the level of internalised stigma they felt around having and desiring condomless sex:

“It feels like a real privilege… and I feel hopeful that you can disassociate yourself from the sense of guilt” (Immediate, high risk, on PrEP, London clinic, aged 30–34).

Reduced anxiety and increased HIV prevention self-efficacy contributed to the lessening of internalised stigma due to putting themselves ‘at risk’ of HIV. For some participants, PrEP also helped to reduce the shame associated with the expectation of external stigma (from friends, prospective sexual partners, and clinicians) as they felt they had been proactive in reducing their HIV risk:

“I think it’s helped that I’ve been on PrEP - I’ve been able to say, I might not use condoms, but I have done something else. I think a lot of what I hear and see in other people is that ‘bad gay’ - it’s the shame that comes with not doing what we are told to do.” (Immediate, low/medium risk, on PrEP, London clinic, aged 30–34).

Others added that non-judgmental support from clinicians had also helped them to accept, be more honest, and feel less ashamed about their sexual behaviour:

“All of a sudden I have this [clinician] who not only seems to understand but also speaks to other people who are in the same situation as me, so I don’t feel alone anymore. I felt so alone before, I felt like I was the only person who felt like this or wanted to do these things, and all of a sudden I have this release, there is a definite case of psychological release with it.” (Deferred, increased risk, on PrEP, London clinic, aged 25–29).

Social norms around the importance of condom use and practicing safer sex within the gay community were repeatedly referred to by the participants. They noted that PrEP disrupted these social norms by removing HIV risk from condomless sex, with one participant describing this as making ‘the unthinkable [desire to have condomless sex], thinkable’. For some, this disruption of social norms had been instrumental in them feeling less shame and guilt around their sexual behaviour:

“Because the person I was before had had bareback sex but it was in a relationship and it was this kind of… enshrined thing, it wasn’t something that was tangible and acceptable it was really taboo and we’ve broken down that taboo now.” (Immediate, high risk, on PrEP, London clinic, aged 30–34).

However, despite the challenge that PrEP presents to existing social norms, many participants continued to describe themselves as ‘promiscuous’ or a ‘slut’ when discussing their sexual behaviour. Although some reclaimed these terms as a way of subverting their power (such as when one participant described himself as ‘a prime example of a Truvada whore’), it was clear that others still perceived having sex with more people as something negative despite enjoying the improved self-efficacy that PrEP has given them:

“PrEP has allowed me to be a bit more I suppose promiscuous but also feeling responsible at the same time and I know that sounds like a double negative.” (Deferred, high risk, on PrEP, Manchester clinic, aged 35–39).

### Increased sexual pleasure and intimacy

Many participants described how they were enjoying sex more as a result of using PrEP. By reducing anxiety and internalised stigma, and increasing self-efficacy, PrEP allowed participants to be more present in their sexual encounters and more focused on their pleasure and that of their partner(s):

“I just enjoyed the moment a lot more and to me it made a difference because it meant that it was a lot less worry for me at the time. I was more concerned about my own partner’s pleasure than I was concerned about what I was going to have to worry about afterwards.” (Immediate, high risk, on PrEP, Sheffield clinic, aged 30–34).

A number of participants commented that PrEP increased intimacy and emotional closeness with sexual partners. For some, this was because they experienced condoms as a barrier to intimacy, and PrEP enabled them to have condomless sex where they would not have done so previously. For others, it was because they were no longer distracted by HIV-related anxiety during sex and were instead better able to focus on being intimate with their partner.

“I think [my sex life] is the best it’s ever been to be honest… it’s the most intimate it’s ever been.” (Deferred, low risk, on PrEP, Manchester clinic, aged 25–29).

A few participants highlighted that they had entered relationships for the first time in their lives since they started using PrEP. While some felt this was coincidental, others credited it to the greater intimacy that they were able to have with sexual partners due to using PrEP.

“I mean maybe I was at a stage in my life anyway where I was ready to try and have a relationship. But I think there was something about the fear and the terror that was taken out of sexual contact that enabled more and repeated intimate contact.” (Deferred, high risk, on PrEP, Sheffield clinic, aged 20–24).

#### Internal conflict

For some participants, it was clear that despite the benefits PrEP provided, it was also a source of internal conflict with a number describing PrEP as a ‘double-edged sword’. While a few attributed this to concerns around STIs and ongoing HIV risk, the remainder appeared to feel conflicted about the sex they were having because they felt using condoms was the ‘ideal’ way to prevent HIV acquisition:

“I don’t know. The ideal I suppose is that we’d be having protected sex [with a condom], regardless. Would I wish for it? I don’t know, I don’t think so.” (Immediate, increased risk, on PrEP, Sheffield clinic, aged 40–44).

“I know [using PrEP] is not necessarily the best way of going about things, but if you’re gonna… put yourself in any danger it’s better to have that extra protection” (Deferred, medium risk, on PrEP, Sheffield clinic, aged 35–39).

A few participants felt that PrEP had contributed to a loss of self-control (in contrast to most participants who experienced an increase in self-efficacy) with regards to condom use or their sexual behaviour. For example, this participant initially described how he felt disempowered by PrEP as the removal of HIV risk made it harder to motivate himself to use condoms, although he later said that ‘disempowered’ was too strong a term:

“I felt disempowered, I felt I’d lost the control… it was becoming a case of the condom use was becoming less and less. I decided that what I needed to do was take myself away so that I can rebuild the thought processes in my head so that I could start to say no I’m going to start to use condoms again.” (Deferred, high risk, on PrEP, Manchester clinic, aged 45–49).

Despite the internal conflict described in this section, the majority of participants who felt this way maintained their PrEP use. Notably, the participant who chose not to take PrEP when offered explained his decision was due to concerns about PrEP stigma and being perceived as someone who engages in ‘high risk behaviour’. A few participants described how this internal conflict led to them considering a break from taking PrEP to compel them to use condoms or reduce their sexual activity, but at the time of interview none had actually gone through with this:

“In some ways I’ve thought to myself maybe I should come off [PrEP], because then I’d have to go back to that chain of thought that I should be using condoms all the time.” (Deferred, high risk, on PrEP, Manchester clinic, aged 45–49).

#### Behavioural impact of PrEP

##### Greater sexual freedom

Approximately half of participants noted that PrEP hadn’t changed their sexual behaviour particularly, it had just made them feel more comfortable and less worried about it. But for some participants, PrEP had enabled them to have sex that they would not have had previously. This included condomless sex with HIV positive partners (who mostly had undetectable viral loads), increasing the frequency and number of their sexual partners, and participating in group sex, amongst other activities. These participants tended to describe these experiences as liberating and fulfilling:

“The benefit was to have uninhibited sex as much as I wanted, where I wanted, when I wanted.” (Immediate, low risk, on PrEP, Manchester clinic, aged 40–44).

“The whole experience has really made me quite I suppose liberated, promiscuous, but also feeling in control and educated” (Deferred, high risk, on PrEP, Manchester clinic, aged 35–39).

Participants also described that by enabling them to have the sex that they wanted, PrEP allowed them to safely pursue their sexual fantasies and desires without putting themselves at risk of HIV:

“I’m having better sex and more of the sex I want. I wanted to explore the wild sex scene that cities like London and Berlin have to offer - but I’m beginning to lose interest in the scene now after about a year and a half… I guess there is a fantasy about quantity but it isn’t always better - but I wanted to try it so I just did it.” (Immediate, increased risk, on PrEP, London clinic, aged 40–44).

However, as noted above, a few participants felt discomfort with the greater sexual freedom that PrEP afforded them, particularly in relation to increased condomless sex.

### Reduced harmful drug use

Just under a third of participants had engaged in chemsex in the 3 months prior to enrolment (see Table 1). In the IDIs, participants were asked more generally about their drug and alcohol use, including during chemsex. Several participants explained that they had previously used drugs and alcohol to reduce their anxiety around condomless sex and repress feelings of shame and guilt. In these cases, participants described their drug use as ‘self-destructive’ or ‘self-harm’. Most described a reduction in harmful drug use due to changes in circumstances and accessing psychological support. However, one participant directly attributed their reduced harmful drug use to PrEP’s removal of anxiety and shame around sex, since it was no longer a prerequisite for them to have uninhibited condomless sex:

“It was easier before to get drunk or take drugs, have unprotected [condomless] sex and then never speak to the person again and feel less able to worry about it, but still worry about it hugely - to then, not feeling anxious around sex, to be able to have intimate sex with people, to not need huge amounts of sex and drugs.” (Deferred, high risk, on PrEP, Sheffield clinic, aged 20–24).

In contrast, participants who did not experience their drug use as harmful reported that their drug use during sex remained the same, with PrEP simply enabling them to reduce their HIV risk while doing so. These participants described how recreational drugs helped them reduce their inhibitions during sex and enjoy increased pleasure and intimacy:

“I nearly always use mephedrone or perhaps occasionally ketamine. It makes me feel very relaxed, sexual, much more intimate with people and less inhibited in my interactions with them.” (Immediate, decreased risk, on PrEP, London clinic, aged 45–49).

However, one participant who had not yet started PrEP expressed concern that he would increase his drug use once PrEP removed the risk of HIV:

“I guess my worry is that PrEP will increase my drug usage… That risk of acquiring HIV limits how often I do [chemsex] right now to maybe once a month or something.” (Deferred, increased risk, not on PrEP, London clinic, aged 35–39).

### More protective sexual health behaviours

Some participants described how the reduction in HIV-related anxiety and internalised stigma following PrEP use helped them develop a healthier, more positive attitude to sex and consequently reduced their sexual activity:

“One of the impacts is that there has been an aspect of normalisation of my sex, which is because it became so stress-free so worry-free, in fact one of the aspects of PrEP is I have less sex, so instead of having these highly problematic sexual behaviours, I still have them, but I think I have calmed down about sex and I have less of them” (Immediate, low/medium risk, on PrEP, London clinic, aged 45–49).

Others noted that PrEP had reduced their use of sex as ‘self-harm’ by breaking the link between sex and danger:

“I used to call it pressing the ‘fuck it’ button, just going off and doing whatever, if I’m doing something for risk… and I did things, like I did do, because I think I’m worthless, then PrEP made that not dangerous, so why bother?” (Deferred, low risk, on PrEP, Manchester clinic, aged 25–29).

Several participants credited the information and counselling they received during clinic visits for improving their confidence about the sex they have and their ability to negotiate condom use:

“I think I’m slightly better than I used to be at using condoms, using them without discussing it, just taking control of that. But I don’t think that’s related to being on the trial or medication - I think it’s from coming [to the clinic] and discussing risks regularly.” (Immediate, increased risk, on PrEP, Sheffield clinic, aged 40–44).

They also felt that the regular visits, risk counselling, and testing supported them to be more proactive in caring for their sexual health:

“PROUD is good because it keeps me having my regular checks - before I joined PROUD, I’d only come to the clinic once a year.” (Deferred, increased risk, not on PrEP, Sheffield clinic, aged 25–29).

“I’m much more aware of the risks now of having unprotected [condomless] sex with people who say they are negative but actually haven’t tested for a long time. I think coming in to see somebody regularly has made me think more about what I do and why I do it.” (Immediate, decreased risk, on PrEP, London clinic, aged 45–49).

## Discussion

This is the first qualitative study of the psychosocial impact of PrEP on gay men in England. Its findings contribute to the growing body of evidence that PrEP can have a substantial positive impact on the health of gay men which goes far beyond its intended clinical purpose of preventing HIV.

A reduction in HIV-related anxiety while using PrEP was a common experience amongst participants. This is consistent with previous research among GBMSM in high income settings, both qualitative [19, 20, 23] and quantitative [21, 22, 27]. In all these studies as well as our own, reduced anxiety appeared to be a result of confidence in the efficacy of PrEP, as well as PrEP providing a greater sense of control over HIV prevention. It is important to note that studies of the first generation of PrEP users tended to attract participants who were at high risk of acquiring HIV and perceived themselves as such, as was the case with the PROUD trial. A more recent Australian study exploring PrEP’s ability to reduce HIV-related anxiety found that this effect may be limited to GBMSM who are clinically assessed as being at high risk of HIV infection [21]. Previous research has also found that some GBMSM have concerns about PrEP use, including fears of being stigmatised as promiscuous or irresponsible, being perceived as HIV-positive, and experiencing side effects [19]. These concerns present important barriers to uptake but, as with the PROUD participants interviewed for our study, they appear to have less influence on PrEP continuation [33–35].

The finding that PrEP can reduce internalised stigma and improve self-efficacy is also supported by previous studies. Collins et al. (2017) identified that the empowering nature of PrEP counteracted feelings of guilt and shame, helping GBMSM using PrEP in Seattle to feel more in control of their HIV risk and overall wellbeing [19]. These qualitative findings were replicated amongst GBMSM participating in the iPREX OLE trial as well as our own study [19, 20]. Quantitative PrEP research has not explored PrEP’s impact on self-efficacy although it has explored similar topics with conflicting results. A Dutch study found PrEP use was associated with a reduction in sexual compulsivity (defined as an individual’s sense that they cannot control their sexual behaviour) [36] while a USA study found no association between PrEP use and sexual self-esteem (defined as self-belief in the ability to protect one’s health) [22]. However, the authors of the USA study felt this may have been due to limitations of the psychological index chosen to measure sexual self-esteem as opposed to a real absence of improvement.

As well as helping users to feel more in control of their health, our findings suggest that PrEP reduces guilt associated with condomless sex by challenging the rationale of social norms pertaining to condom use. However, they also highlight the resilience of these norms and their influence on PrEP stigma. Race (2015) argued that PrEP’s promise in eliminating HIV risk could be undermined by its challenge to existing social norms that had previously served (some of) the gay community well [37], and this appears to be reflected in the experiences of internal conflict and reduced self-efficacy among some PROUD participants. Although some participants experienced internalised PrEP stigma and some anticipated stigma from others, there were limited reports of external PrEP stigma, in contrast to PrEP users in the USA and Canada interviewed in the same period [19, 38]. This may be due to PROUD participants being among the first PrEP users in the UK and interviews being conducted prior to the controversy surrounding NHS commissioning, upon which PrEP gained national attention [39]. A 2019 cross-sectional survey of UK PrEP users found that 17% felt they had been treated differently because of their PrEP use, with open text responses indicating they had experienced slut-shaming or HIV stigma (as they had been assumed to be living with HIV) [27].

PrEP’s influence on social norms is likely to be complex and dynamic over time as it becomes a more established method of HIV prevention. Indeed, while PrEP contradicts certain social norms, it may reinforce others. The increased self-efficacy and reduced self-stigma experienced by participants could be a consequence of PrEP enabling them to more fully participate in the neoliberal ‘politics of homonormativity’ which reduces HIV prevention to a matter of personal responsibility [40, 41]. More recent research from Australia has produced evidence of stigmatisation of non-use of PrEP among GBMSM, with PrEP replacing condoms as the new ‘safe sex orthodoxy’ [42]. This development suggests that HIV prevention services in the UK should be careful to communicate PrEP as being one choice in a range of efficacious and acceptable HIV prevention options if the reduced shame and guilt experienced by PrEP users is not simply to be transferred to those not using PrEP [42].

Several participants credited sexual counselling and the opportunity to speak openly about their sex lives with non-judgemental and supportive clinicians with reducing the shame they felt about their sexuality and sexual behaviour, in addition to taking PrEP itself. Similarly, the AmPrEP study reported difficulties in disentangling the impact of PrEP on reduced internalised stigma from the general benefits of participating in a study where non-judgemental support and counselling was provided [36]. Previous research indicates that PrEP should be delivered within a holistic intervention which addresses other health needs of the targeted population in order to support uptake and adherence [43]. For GBMSM, it is clear from our study that creating space for supportive and non-judgmental conversations about sex is an important aspect of a holistic PrEP intervention to support sexual wellbeing.

Previous qualitative research has reported that GBMSM experienced an increase in sexual pleasure and intimacy in their relationships as a result of using PrEP [19, 23]. Notably among the PROUD participants, this included men who had previously only engaged in casual sex, who felt that PrEP allowed them to have healthier, more committed relationships as they no longer feared or felt guilty about the sex they were having. Most of these participants reported that they had reduced their sexual activity and harmful drug use – changes which were also identified and captured quantitatively in the AmPrEP study [36]. However, as demonstrated in previous analysis of the PROUD study, PrEP’s impact on sexual behaviour is diverse and may fluctuate over time depending on the individual’s circumstances [32] – other participants reported an increase in sexual activity and continued drug use while using PrEP. What draws these diverse experiences together is that fear of HIV transmission no longer factors in these decisions, allowing more room for consideration of personal wellbeing instead.

This analysis provides an important baseline for researchers to understand the psychosocial impact on gay men in England who were amongst the first to receive PrEP within the formal healthcare system, allowing for any changes over time to be accurately captured. It also provides further detail to support quantitative researchers to develop more accurate measures of PrEP’s psychosocial impact, including on social norms, to study trends and interactions between outcomes.

This analysis was constrained by the demographics of those who participated in the PROUD trial. IDI participants were predominantly white, gay cisgender men with high levels of education and in full-time employment – bisexual and other men who have sex with men, trans GBMSM, GBMSM of colour and those from lower socio-economic backgrounds are likely to have different or additional experiences to those documented here. In addition, PROUD study participants generally were, and perceived themselves as being, at high risk of acquiring HIV, meaning these findings may not apply to gay men who do not perceive themselves as being at such high risk. Some key populations at risk of HIV in the UK, such as heterosexual Black African men and women and people who inject drugs, were not eligible to participate in the trial. Although the trial eligibility criteria included trans women, only three enrolled in the trial and only one was interviewed, limiting insight into trans women’s experience of using PrEP. Given that trans women bear a disproportionate burden of HIV and are underrepresented in HIV prevention research [44], this is a significant limitation to the study. The Impact trial, an implementation study conducted in England subsequent to PROUD, has reported that, at baseline, 4.38% of its participants (1038 out of 24,255) are not MSM, including 359 trans women, that 8% of all participants were from Black or Asian ethnic groups, and 40% were born abroad [45]. This presents a potential opportunity to explore psychosocial impact among groups within and beyond gay men.

Future quantitative research into PrEP should consider including measures of the psychosocial impact of PrEP as this will support understanding of its scale, who is most likely to benefit, and any trends over time (particularly in relation to changing social norms). The introduction of routine commissioning of PrEP on the NHS in England in October 2020 offers an exciting opportunity to study the psychosocial impact of PrEP on a much larger scale.

Our findings support calls to consider the psychosocial impact of PrEP in prescribing guidelines, particularly where patients are likely to gain psychosocial benefits from its use. Such considerations are largely absent from national PrEP guidelines, both in the UK and elsewhere. One exception is Australia, where guidelines allow for clinical discretion in prescribing PrEP to patients experiencing severe HIV-related anxiety [46]. Although WHO guidelines make no reference to HIV-related anxiety, they do suggest that an expressed need for PrEP should be considered a legitimate indication for PrEP prescription [47]; e.g., South Africa has done this by including “requesting PrEP” as an eligibility criterion [48]. In contrast, PrEP eligibility criteria for England (and the other devolved nations of the UK) are all determined through clinical assessment of HIV risk [49–52].

Research into PrEP provider perspectives on prescribing PrEP for HIV-related anxiety indicates that there is support for prescribing PrEP where HIV-related anxiety is aligned with HIV risk, but clinicians may feel less comfortable prescribing PrEP to those they perceive as the ‘worried well’ [53–56]. Clinician discomfort may be countered through guidelines which highlight that those who express a need for PrEP may not be disclosing their true level of risk for a variety of reasons, including fear of judgement or simply not being ready to discuss certain issues with their clinician [55]. In addition, adopting a broader definition of sexual wellbeing which goes beyond a narrow biomedical focus on risk reduction in clinical guidelines would emphasise that mental distress should be taken as seriously as risks to physical health [57].

Simultaneously, it is important to acknowledge concerns around the overmedicalisation of psychosocial issues such as HIV-related anxiety, and how a reliance on biomedical interventions may lead to the neglect of other psychosocial interventions [58]. Indeed, a number of participants in this analysis attributed psychosocial improvements to having the opportunity to engage in non-judgemental discussion with clinicians about sex and HIV, in addition to PrEP itself. Rather than being a case of either/or, use of PrEP may free up the mental space required to address deeper psychosocial issues, since the immediate fear of acquiring HIV is removed. Consequently, it is important that as PrEP becomes part of established service provision in the UK, it remains part of a wider package of HIV prevention tools, including HIV education and psychosocial support such as sexual counselling, chemsex support, and mental health services. This is especially important given that some gay men may struggle with internal conflict and reduced self-efficacy in response to their PrEP use.

## Conclusion

In summary, this analysis suggests that by removing HIV risk from sex, PrEP improves users’ wellbeing by reducing HIV-related anxiety and internalised stigma and increasing HIV prevention self-efficacy, sexual pleasure, and intimacy. In turn, these psychological changes may influence behaviour in the form of greater sexual freedom, reduced harmful drug use, and more protective sexual health behaviours. However, PrEP may create internal conflict for some gay men, due to its disruption of social norms around condom use and its perceived influence on their sexual behaviour leading to reduced condom use self-efficacy. Our findings support calls to consider the psychosocial impact of PrEP in prescribing guidelines. They also highlight the need for prescribers to be aware of the risk that some PrEP users may experience internal conflict and reduced self-efficacy around condom use, and to be able to offer psychosocial support as part of the PrEP service package. These findings provide a baseline of PrEP’s psychosocial impact amongst some of the first PrEP users in England and could potentially inform current and future PrEP provision and research in a range of populations and settings.

## Data Availability

The PROUD data are held at MRC CTU at UCL, which encourages optimal use of data by employing a controlled access approach to data sharing (http://www.ctu.mrc.ac.uk/our_research/datasharing/). All requests for data are considered and can be initiated by contacting mrcctu.ctuenquiries@ucl.ac.uk or through the URL: http://www.ctu.mrc.ac.uk/our_research/datasharing/application_process/. The basis for this project originated from a MSc research project undertaken by RHa at the London School of Hygiene & Tropical Medicine (LSHTM), and ethical approval for this MSc project was granted by LSHTM. Data was accessed from University College London (UCL) and the Medical Research Council (MRC) Clinical Trials Unit (CTU) through a clinical data disclosure agreement and PROUD sub-study proposal agreement.
